# Glycoproteins Involved in Sea Urchin Temporary Adhesion

**DOI:** 10.3390/md21030145

**Published:** 2023-02-24

**Authors:** Inês Ventura, Victoria Harman, Robert J. Beynon, Romana Santos

**Affiliations:** 1 Centro de Ciências do Mar e do Ambiente (MARE), ARNET—Aquatic Research Network, Departamento de Biologia Animal, Faculdade de Ciências, Universidade de Lisboa, 1749-016 Lisboa, Portugal; 2 Centre for Proteome Research, Institute of Systems, Molecular and Integrative Biology, University of Liverpool, Liverpool L69 7ZB, UK

**Keywords:** sea urchin, tube feet, bioadhesive, glycoproteins, biomimetic adhesive

## Abstract

Biomedical adhesives, despite having been used increasingly in recent years, still face a major technological challenge: strong adhesion in wet environments. In this context, biological adhesives secreted by marine invertebrates have appealing characteristics to incorporate into new underwater biomimetic adhesives: water resistance, nontoxicity and biodegradability. Little is still known about temporary adhesion. Recently, a transcriptomic differential analysis of sea urchin *Paracentrotus lividus* tube feet pinpointed 16 adhesive/cohesive protein candidates. In addition, it has been demonstrated that the adhesive secreted by this species is composed of high molecular weight proteins associated with N-Acetylglucosamine in a specific chitobiose arrangement. As a follow-up, we aimed to investigate which of these adhesive/cohesive protein candidates were glycosylated through lectin pulldowns, protein identification by mass spectroscopy and in silico characterization. We demonstrate that at least five of the previously identified protein adhesive/cohesive candidates are glycoproteins. We also report the involvement of a third Nectin variant, the first adhesion-related protein to be identified in *P. lividus*. By providing a deeper characterization of these adhesive/cohesive glycoproteins, this work advances our understanding of the key features that should be replicated in future sea urchin-inspired bioadhesives.

## 1. Introduction

Biomedical adhesives have been increasingly used over the past years, exhibiting attractive features for an array of medical applications. However, achieving strong adhesion in wet environments is still a technological challenge. In this context, biological adhesives secreted by marine invertebrates have three appealing characteristics to incorporate into new underwater biomimetic adhesives: water resistance, nontoxicity and biodegradability. Nevertheless, only a very limited number of marine invertebrates have been used as models.

The best-characterized system is the mussel byssal plaque, in which DOPA (3,4-dihydroxyl-phenylalanine), a chemical group formed by post-translational modification of tyrosine residues, plays important interfacial adhesive and bulk cohesive roles [1]. Most of the biomimetic adhesives developed so far thus rely on DOPA chemistry [1,2]. More recently, other organisms relying on permanent adhesion, such as tubeworms and barnacles, and temporary adhesion, such as sea stars, have also been used as models since their adhesive protein sequences have become available [3,4,5]. In addition to L-DOPA, which was only described in permanent adhesion, protein post-translational modifications (PTMs) might also play a role in wet adhesion [6]. Glycosylation, in particular, is highly prevalent in aquatic adhesives, in both permanent and temporarily attaching organisms [7,8,9]. The roles of glycosylation in marine adhesive proteins are still speculative but have been proposed to increase conformational stability, enhance protein binding ability, and make proteins more resistant to degradation [8,10,11,12,13,14].

Echinoids, which rely on temporary adhesion to survive, possess a specialized adhesive organ—the tube feet. Remarkably constant for all echinoderm species, it consists of (1) an enlarged and flattened distal extremity (disc), which contacts the substratum, and a proximal extensible cylinder (stem) that connects the disc to the test. Functioning as a duo-gland adhesive system, the adhesive cells release a proteinaceous secretion, while the de-adhesive cells secrete a releasing secretion allowing tube foot detachment [15]. During the adhesive process, adhesive secretory granules are extruded at the tip of microvillar-type cell projections, which are arranged in a tuft at the cell apex. The adhesive cell granules vary extensively from one echinoderm taxon to another, both in terms of size and internal ultrastructure. These secretory granules are usually made up of at least two materials of different electron densities which gives them a complex ultrastructure [15]. Our lab updated the last model proposed for sea urchin temporary adhesion [16], demonstrating the involvement of a large glycoprotein with N-acetylglucosamine (GlcNAc) oligomers and two smaller proteins with terminal N-acetylgalactosamine (GalNAc) in *Paracentrotus lividus* adhesion [17]. Using *Lycopersicon esculentum* (tomato) lectin (LEL) histochemistry, it was found that the amino sugar N-acetylglucosamine is restricted to the outer ring of spherical structures, most likely corresponding to the heterogenous adhesive secretory granules inside the cell ducts, that end in apical tufts at the disc cuticle. These results, combined with lectin blots, indicated the presence of a high molecular weight glycoprotein (>180 kDa) with N-acetylglucosamine in the form of chitobiose [GlcNAcβ(1,4)GlcNAc], most likely four units, in the outer rim of the more abundant adhesive granules, as well as in the less abundant homogenous adhesive granules. Due to its specific localization and presence in the adhesive footprint, we proposed this glycoprotein as the main component of *P. lividus* adhesive secretion [17]. N-acetylgalactosamine conjugated with two glycoproteins (apparent molecular weight of 72 and 135 kDa) was also detected in the adhesive epidermis and the footprint, labeling what seems to be the microvilosities and cytoplasm of abundant epidermal support cells [17]. Despite not being directly involved in adhesion, these proteins can still indirectly affect adhesion as shown in flatworm temporary adhesion [8]. These results are further supported by research on other echinoderm adhesives, such as sea stars, *Asterias rubens*, where two high molecular weight glycoproteins (Spf-290 and Spf-210) contain N- and O-glycans with terminal fucose linked to galactose residues, sialic acids linked to N-acetylgalactosamine residues, and terminal N-acetylgalactosamine linked to galactose residues. The footprints (i.e., the material that is left on the substrate upon detachment) also contain large glycoconjugates with sialic acids [14]. These results are further supported by the high expression of hydrolases acting on glycosyl groups such as asparaginase-like proteins in sea urchin adhesive disc, proposed as a component of their de-adhesive secretions [18].

To date, a total of 16 putative adhesive/cohesive proteins were pinpointed in the sea urchin *P. lividus*, based on their similar in-situ hybridization expression, specifically labeling the location of the adhesive cells, together with their putative functions based on the obtained BLAST hits and domain prediction [19]. Six of these (Nectin, Alpha-tectorin, Uncharacterized protein, Myeloperoxidase, Neurogenic locus notch homologous protein and Alpha-macroglobulin) share significant sequence similarity with sea star orthologue adhesion-related genes [19], mainly due to the presence of protein domains recurrently described as temporary adhesive building blocks [4]. The identification of several proteins involved in sea urchin adhesives is further corroborated by the multi-proteinaceous nature of other temporary adhesives from sea stars [20] and flatworms [8]. All six *P. lividus* transcripts had predicted glycosylation sites and signal-peptide sequences compatible with their location in the secretory adhesive cells [19]. 

Sugar moieties are present in the adhesive secretory cells and/or adhesive material of other reversibly-attaching aquatic animals, not restricted to Echinodermata. In flatworms such as *Macrostomum lignano*, the adhesive protein (Mlig-ap2) is glycosylated [8,21,22,23] and it was visualized in the adhesive granules and the footprint using peanut agglutinin lectin (PNA, that binds, with high specificity, the sugar galactosyl (β-1,3) N-acetylgalactosamine) histochemistry [5,8]. Similar results were obtained in other species, such as the proseriate *M. ileanae* [24]. Glycosylated proteins were also reported in the footprint meshwork and in the secretory cells of the cnidarian *Exaiptasia pallida* [25], a finding supported by transcriptomic studies [26,27]. Consistently, with platyhelminths (*M. lignano* and *M. ileanae*) and anemones (*E. pallida*), PNA has also been reported as a novel adhesion marker in tunicate larvae (*Ciona intestinalis*) [9]. Sialylated N-glycans with terminal galactoses and N-acetylglucosamines are also involved in *C. intestinalis* adhesion [9]. 

Despite knowing that many candidate adhesive proteins are glycosylated or contain predicted glycosylated residues and the possible role of glycosylation in marine adhesive proteins, Nectin was the only sea urchin adhesive/cohesive protein [18] proven to have several glycosylated isoforms [28,29,30,31]. Therefore, as a follow-up, the present work aims to pinpoint within the 16 adhesive/cohesive protein candidates previously identified [19], those which are conjugated with GlcNAc and GalNAc, since these glycans are known to be present in sea urchins’ adhesive granules [17,32]. For this, we performed glycoprotein pulldown assays using agarose beads conjugated with three lectins that bind different arrangements of GlcNAc (GSL II, WGA, LEL) and one lectin that binds GalNAc (SBA), followed by protein identification by mass spectrometry and in silico characterization. This approach confirmed that 5 out of the 16 sea urchin adhesive/cohesive candidates are glycoproteins. By providing new evidence regarding the multiprotein complexes involved in sea urchin temporary adhesion and its molecular features, this work provides a step towards understanding the key features that should be mimicked in future sea urchin-inspired bioadhesives.

## 2. Results

### 2.1. Adhesive Proteins Pulldown Assays

Lectin pulldowns were performed to obtain fractions enriched in glycoproteins associated with the glycans of interest, taking advantage of the reversible and highly selective binding ability of lectins to mono- and oligosaccharides. Non-binding proteins (that do not bear these specific glycans) were collected in the unbound fraction (UB). Agarose beads were thoroughly washed and further eluted, ensuring that the eluted fraction is composed only of the proteins containing the glycans of interest. Inhibited pulldowns (using agarose beads whose bounded lectins were previously incubated with its binding sugars prior to incubation with protein extracts) were also performed as a negative control. 

**Figure 1:**
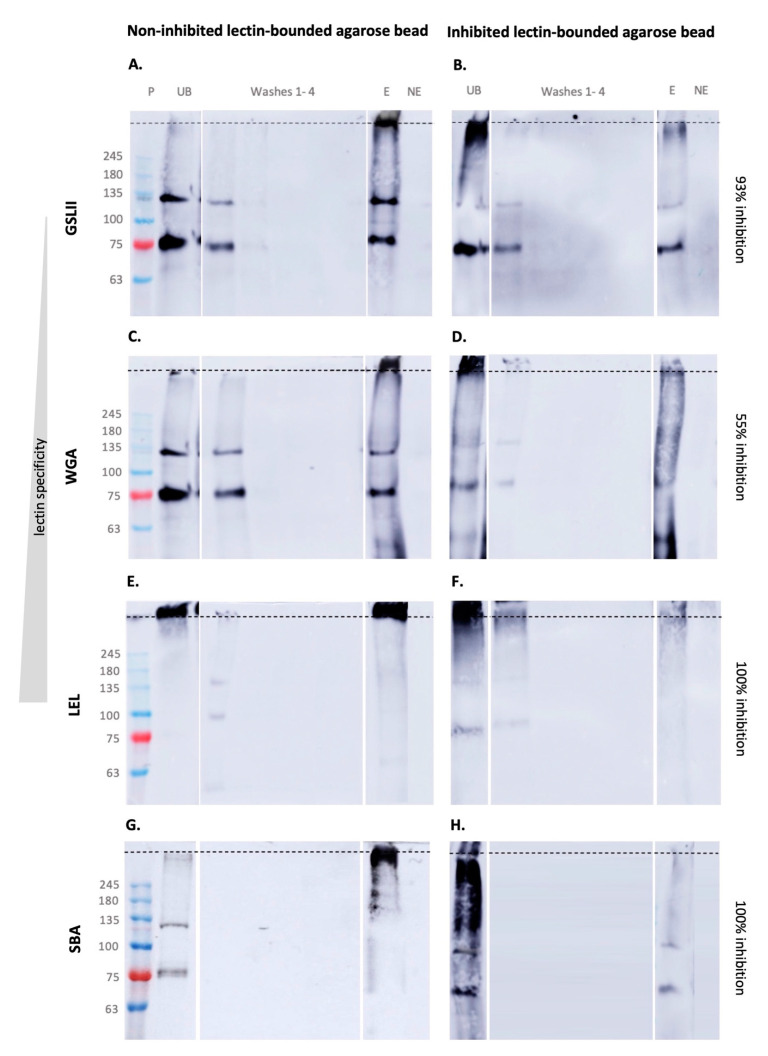
Glycoproteins pulldown assay from *Paracentrotus lividus* tube feet discs protein extracts using lectin-bounded agarose beads. Lectin blot of pulldowns using non-inhibited (**A**,**C**,**E**,**G**) and inhibited (**B**,**D**,**F**,**H**) lectin-bounded agarose beads. Abbreviations: E—eluted fraction; M—molecular weight markers; NE—non-eluted fraction; UB- unbound fraction; Wash 1–4—wash fractions. The dashed line indicates the separation between stacking and resolving gel. GSL II, *Griffonia simplicifolia* lectin II; WGA, Wheat germ agglutinin; LEL, *Lycopersicon esculentum* lectin; and SBA, Soybean agglutinin.

*Griffonia* (Bandeiraea) *simplic*ifolia Lectin II (GSL II), which detects N-acetylglucosamine (GlcNAc), was previously described to detect glycoproteins in the tube feet discs, with apparent molecular weights of 72 and >180 kDa. GSL II labeled the disc cuticle and adhesive epidermis, as well as the secreted adhesive [17]. We detected intense bands around 75, 135 and >245 kDa in the eluted fraction. GSL-II labeled proteins (75 and 135 kDa) were also visible in the unbound fraction (UB) and initial wash fraction (W1), indicating a higher amount of those proteins (Figure 1A). 

Wheat germ agglutinin (WGA), which detects one or two chitobiose units, was previously reported to label the adhesive epidermis and cuticle in tube feet discs, as well as the adhesive footprints. In tube feet discs, it also detects glycoproteins with an apparent molecular weight of 35, 75, 135 and >180 kDa [17]. In the eluted fraction, we detected an intense band at >245 kDa, and two less intense ones around 75 and 135 kDa. Similar to GSL II, intense bands around 75 and 135 kDa were detected with WGA in the unbound fraction, showing an increase in the specificity of the pulled-down proteins (Figure 1C). Reinforcing this result, LEL-labelled proteins (targeting specifically a higher number of chitobiose units, previously shown to be present in adhesive granules, expressed in the adhesive epidermis and secreted into the adhesive footprints [17]) were only detected above 245 kDa (Figure 1E). These results confirm the presence of high molecular glycoproteins with *n* ≥ 4 chitobiose residues, so large that they fail to enter the resolving gel. A band at the same molecular weight was detected in the UB fraction, possibly reflecting the need for a higher volume of beads to capture all LEL-labelled proteins present in the disc. 

Inhibited pulldowns (accessing lectin/glycan selective binding) resulted in a clear decrease in terms of the protein amount and the number of protein bands detected in the eluted (E) fraction of the inhibited pulldown, in comparison with the non-inhibited one (Figure 1B,D,F).

WGA inhibition was partially effective, as demonstrated by the presence of several proteins in the eluted (E) fraction, above 48 kDa (Figure 1D). Some glycoproteins, probably with many chitobiose residues, were able to attach to the beads in the eluted fraction (E). However, an intense band in the stacking gel was detected in the UB fraction, indicating that the binding of the above-mentioned protein with >245 kDa was successfully inhibited. The same results were obtained for GSL II (Figure 1B) (higher amounts of GlcNac-containing proteins were detected, with a defined band in the eluted fraction). LEL presented a total inhibition (Figure 1F). 

Soybean agglutinin (SBA) lectin was previously described to detect two glycoproteins in *P. lividus* tube feet discs, with an approximate molecular weight of 75 kDa and 135 kDa. This lectin, which detects N-acetylgalactosamine, labeled the adhesive footprint and the disc adhesive epidermis, targeting microvilosities and the cytoplasm of abundant epidermal cells—most likely support cells [17]. Our results show that this glycan is also associated with a high molecular weight protein (>245 kDa). Proteins with lower molecular weight (around 75 and 135 kDa), possibly containing fewer GalNAc residues, could not bind were detected in the UB fraction (Figure 1G). SBA inhibition was substantial since only two faint bands could be visualized in the eluted (E) fraction (Figure 1H).

No glycoproteins were detected in the non-eluted (NE) fraction of all pulldowns, indicating the successful recovery of all glycoproteins of interest. Inhibition differences can be related to the capacity of the used sugar solution to inhibit the different lectins.

### 2.2. Lectin Inhibition Assessment

Two sugar mixtures reported to elute glycoproteins from agarose-bound N-acetylglucosamine- or chitin-binding lectins and from agarose-bound Galactose/GalNAc binding lectins were used to inhibit the previously-presented pulldowns. 

**Figure 2:**
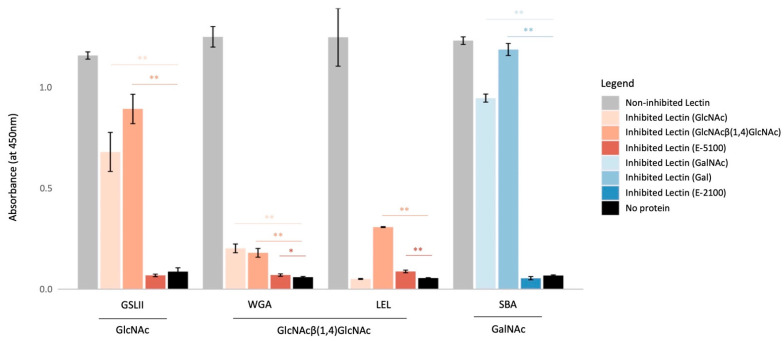
Lectin inhibition comparative analysis. Enzyme-linked lectin assay comparing the obtained absorbance values for non-inhibited and inhibited lectins. Assay performed in a 96 wells plate coated with *Paracentrotus lividus* tube feet discs protein extracts. The legend shows the tested inhibiting sugar or sugar solution. Black bars are controls with no bound protein. GSL II was used to detect the presence of N-acetylglucosamine; WGA and LEL to detect chitobiosis and SBA to detect N-acetylgalactosamine in the extracts. E-5100 glycoprotein eluting solution is comprised of GlcNAc and chitin, while E-2100 contains galactose and GalNAc. Each bar represents the mean and the standard deviation (*N* = 8). Parametric T-tests were performed to compare absorbance values of each lectin; * *p* < 0.05 and ** *p* < 0.001. Abbreviations: GSL II, *Griffonia simplicifolia* lectin II; Gal, galactose; GalNAc, N-acetylgalactosamine; GlcNAc, N-acetylglucosamine; LEL, *Lycopersicon esculentum* lectin; SBA, Soybean agglutinin; WGA, Wheat germ agglutinin.

An enzyme-linked lectin assay (ELLA) was performed to verify the efficiency of the inhibition solution used. We compared absorbance values for non-inhibited lectins (represented in grey Figure 2), lectins inhibited with simple sugars (GlcNAc, GlcNAcβ(1,4)GlcNAc, GalNAc, Gal) (lighter colors), lectins inhibited with a mixture of sugars (darker colors) and a negative control (where no protein extract was added) (black). 

By comparing to previously obtained results [17], we verified that using a sugar solution instead of simple sugars improves the inhibition of GSL II, WGA and SBA (Figure 2, Tables S1 and S2).

Similar to previous results, galactose (Gal) did not inhibit SBA; WGA was inhibited with N-acetylglucosamine (GlcNAc) and LEL with chitobiose (GlcNAcβ(1,4)GlcNAc) [17]. GSL II was also inhibited with GlcNAcβ(1,4)GlcNAc. Except for SBA, lectin inhibition with both single or a mixture of sugars significantly decreased the measured absorbance (Figure 2). However, the use of a complex sugar solution (that includes the two single sugars tested separately for each lectin) fully inhibited GSL II, WGA and SBA. The results obtained were similar to the negative control (Table S2). LEL, however, was fully inhibited by the simple sugar—GlcNAc, but not with the sugar mixture, indicating that the chitin/GlcNAc proportion used in the complex solution decreases LEL inhibition. 

### 2.3. Identification of the Pulled-Down Glycoproteins

Each glycoprotein eluted fraction (see Figure 1) obtained with lectin pulldown assays was further analyzed by tandem mass spectrometry (MS/MS) (see Proteomic workflow in Figure S1, supplementary material). For GSL II, we obtained 1250 protein hits (i.e., 1250 proteins found in the sample), followed by WGA (1222 hits) and LEL (1189 hits). A total hit of 1074 proteins was obtained for SBA. 

**Figure 3:**
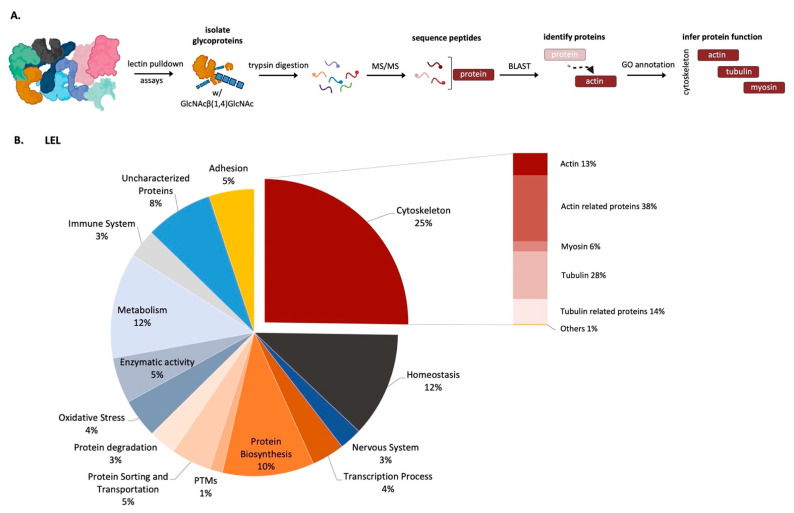
Global protein composition by functional groups of LEL-pulldown eluted fraction. Experimental approach used to identify proteins pulldown with LEL-bounded agarose beads (**A**) and the correspondent GO annotation analysis of *Paracentrotus lividus* disc proteins conjugated with GlcNAcβ(1,4)GlcNAc (**B**).

A computational analysis of the acquired MS/MS data using BLAST allowed protein identification while accessing their potential functionality according to their GO annotations (Table S3). In accordance with [18], the pulldown proteins are involved in cytoskeleton organization, metabolism, secretory pathways, transcription/translation processes, and protein biosynthesis and putative components of the adhesive secretions (Figure 3, Table S3). These results were similar in the four tested lectins (Figure S2). 

Cytoskeleton-related proteins represented the largest fraction of the proteins bound to each lectin. These included actins, myosins and tubulins, and other proteins known to directly interact with them (Figure 3). 

**Figure 4:**
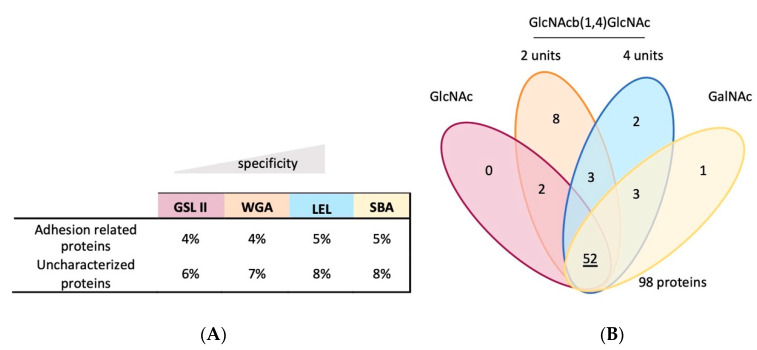
Adhesion-related and uncharacterized *Paracentrotus lividus* tube feet disc glycoproteins. Percentage of adhesion-related and uncharacterized proteins recovered in the different glycoprotein pulldown assays (**A**) and their conjugated glycans (**B**).

LEL and SBA pulled down the highest percentage of adhesion-related proteins (proteins with a GO annotation related to adhesion and the prediction of domains recurrent in temporary adhesive proteins) and uncharacterized proteins (Figure 4, Table S3). A more detailed analysis showed that only a few of those proteins were exclusively associated with one type of glycan, indicating that the pulled-down proteins were the most likely to possess multiple glycosylation sites and different glycan residues. 

### 2.4. Selection of Adhesive/Cohesive Candidate Glycoproteins

To focus the analysis on potential adhesive/cohesive glycoproteins, the 16 candidate proteins shown by [19] to have an in situ hybridization pattern coincident with adhesive cells in *P. lividus* were searched in our dataset of putative adhesion-related (34 proteins) and uncharacterized proteins (64 proteins) (Table S3). Of these, six were present in all four lectin pulldowns: Nectin, Alpha-tectorin-like protein, Myeloperoxidase, Alpha-2-macroglobulin-like protein 1, and two Uncharacterized proteins (Table 1 and Table S4). All, except for transcripts TR46688_c0_g1_i1 and TR46467_c1_g1_i2 (i.e., Uncharacterized proteins), have adhesion-related sea star orthologous genes. 

Our new BLAST analyses improved the annotation of *P. lividus* candidate adhesive/cohesive proteins (i) by identifying transcripts previously described as different proteins (transcripts TR63383_c2_g1_i1 and TR46688_c0_g1_i1) that correspond to two segments of a large Alpha-tectorin-like protein, homologous to an Uncharacterized protein LOC100892803 from the sea urchin *Strongylocentrotus purpuratus*; and (ii) by establishing the correspondence between transcript TR46467_c1_g1_i2_6, which had no former known homology, to the Uncharacterized protein LOC115927989 from the sea urchin *S. purpuratus*.

Based on the sequence coverage (~35% and 40 unique peptides, Table 1), Nectin is present in high abundance in all pulldowns (Table 1). It is a large protein (108 kDa) with no predicted N-glycosylation sites, but with 11 predicted O-glycosylation sites (Figure S3, Table S5). A lower number of unique peptides was obtained for Alpha-tectorin-like protein (TR63383_c2_g1_i1_5 and TR46688_c0_g1_i1_6) and Alpha-2-macroglobulin protein (TR61622_c8_g1_i2_4) (Table 1), which can be explained by the large size of these two proteins and the low number of O-glycosylation sites of Alpha-2-macroglobulin (Table S5). Based on the sequence coverage and the number of unique peptides obtained in each lectin pulldown assay, it seems that Alpha-tectorin has a higher number of chitobiose units [(GlcNAcβ1,4)n, n ≥ 4]; detected by LEL] and GalNAc residues (detected by SBA), since lower coverage was obtained in WGA-bounded agarose bead pulldowns (specificity towards (GlcNAcβ1,4)n, n ≤ 2). Alpha-2-macroglobulin appears to have a similar amount of the different sugar residues (Table 1 and Tables S7 and S8). The lowest number of unique peptides was registered for both Myeloperoxidase and the Uncharacterized protein LOC 115927989 (Table 1). Based on sequence-derived predictions, this result reflected a lower protein abundance (Myeloperoxidase) and a lower extent of glycosylation (Uncharacterized protein) (Tables S5, S9 and S10). 

### 2.5. Adhesive Candidate Characterization

**Figure 5:**
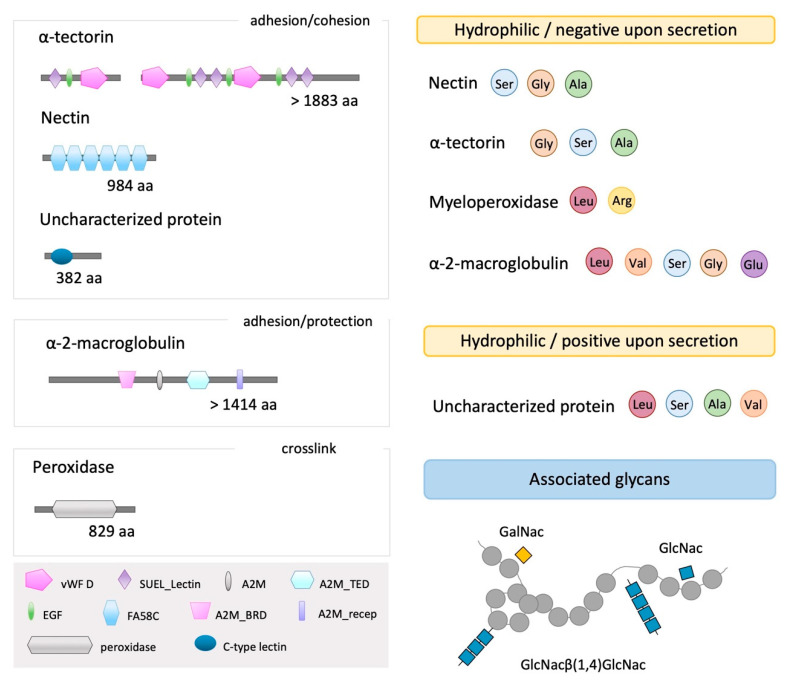
Summary of the characterization of glycosylated adhesive/cohesive protein candidates. Abbreviations: A2M, alpha macroglobulin domain; A2M_BRD, alpha macroglobulin bait region; A2M_recep, alpha macroglobulin receptor binding domain; A2M_TED, alpha macroglobulin receptor binding domain; Ala, alanine; Arg, arginine; EGF, EGF-like calcium-binding domain; FA58C, discoidin domains; GalNAc, N-acetylgalactosamine; GlcNAc, N-acetylglucosamine; GlcNAcβ(1,4)GlcNAc, N-acetylglucosamine in a specific chitobiose arrangement; Gly, glycine; Glu, glutamine; Leu, leucine; Ser, serine; SUEL_Lectin, SUEL lectin domain; Val, valine; vWF D, von Willebrand factor type D domain.

Using bioinformatic analysis, several parameters were analyzed: (1) sequence completeness; (2) presence of a signal peptide (a secretory pathway marker); (3) cysteine content (intra- and intermolecular disulfide bonds have been indicated as responsible for the insolubility of the adhesive [6]); (4) predicted glycosylation; (5) conserved domains, to search for sequence similarity and temporary adhesion related functional building-blocks; (6) molecular weight; and (7) the isoelectric point that denotes protein charge upon secretion (pH increase between the secretory granule and seawater has been proposed as a trigger for bioadhesive polymerization [19]) (Figure 5, Table 2 and Table S11).

Nectin is a 984 amino-acid long protein, with a predicted signal peptide and six tandemly repeated discoidin domains (FA58C, coagulation factor 5/8 C-terminal domain). To date, Nectin is the only confirmed adhesive/cohesive protein in sea urchins, for which two variants have been already described (Uniprot Q70JA0 and A0A182BBB6) [18,28,29] and that has also a sea star orthologue gene (Arub-27). TR60905_c1_g1_i1_5 presents 99.1% similarity with Nectin variant 2 (Uniprot A0A182BBB6) and 98.4% with Nectin variant 1 (Uniprot Q70JA0), indicating that it might correspond to a 3^rd^ variant. All variants presented a predicted signal peptide, followed by six tandemly repeated discoidin domains (FA58C). An LDT sequence (Leu-Asp-Thr) proposed as an integrin receptor binding site by [29], was present in all sequences in FA58C II. Serine (Ser), glycine (Gly) and alanine (Ala) are the most abundant amino acids in all variants (Table S11). Predicted to be thermos- and structurally stable, all variants are also predicted to be hydrophilic and negatively charged upon secretion into seawater. The predicted molecular weight (approximately 108 kDa) is concordant with previous research [17,29,30].

Alpha-tectorin like is a >1873 amino-acid long protein with several conserved domains organized as modules; five SUEL lectin domains (SUEL_Lectin), four calcium-binding epidermal growth factor-like domains (EGF) and three von Willebrand type D domain (vWD). Reporting 77.1% (TR63383_c2_g1_i1) and 25.89% (TR46688_c0_g1_i1) similarity with sea urchin *S. purpuratus* Uncharacterized protein LOC100892803 (NCBI XP_030852014.1), an Alpha-tectorin like protein, we demonstrated that these transcripts correspond to different segments of an Alpha-tectorin-like protein (named as consensus sequence) (Figure S4). Despite sharing partial sequence homologies with Alpha-tectorin (50% identity), sea star *A. rubens* Spf1 did not align well with both Uncharacterized proteins (NCBI XP_030852014.1 and our consensus sequence) and therefore only its molecular features were considered for comparison purposes (Table S11). *S. purpuratus* and *P. lividus* sequences did not have a predicted signal peptide. However, since Spf1, Mlig-ap1 and Mlig-ap2—all Alpha-tectorin-like proteins—are predicted to be secreted and no start codon was found for the sea urchin sequences, the possibility that sea urchin Alpha-tectorin might also be secreted cannot be excluded. With an amino acid bias towards glycine (Gly), serine (Ser) and alanine (Ala), Alpha-tectorin-like proteins are highly hydrophilic (negative GRAVY) (Table S11). Only Spf1 was predicted as stable, showing the lowest instability index (Table S11). With a theoretical pI around 4.5–5, they are most likely negatively charged upon secretion into seawater. With a high molecular weight (>200 kDa), these proteins are predicted to be highly glycosylated, containing both N- (most likely chitobiose units up to four units) and O- (GalNAc residues) glycans. 

Alpha-2-macroglobulin is a >1414 amino-acid long protein with several conserved domains: an alpha-2-macroglobulin bait region (A2M_BRD), followed by an alpha-2-macroglobulin (A2M), an alpha-2- macroglobulin thiol ester-containing domain (A2M_TED) and an alpha-2-macroglobulin receptor binding domain (A2M_recep). Sequence alignment with sea urchin *S. purpuratus* Alpha-2-macroglobulin-like homologous protein (NCBI XP_011676138.2, 69.86% similarity) showed that (1) the 5′ end of TR61622_c8_g1_i2 transcript was not sequenced in the tube foot transcriptome and that (2) there is a possible interspecific difference regarding the size of these proteins (Figure S3). A signal peptide is only predicted in *S. purpuratus* Alpha-2-macroglobulin-like protein, however, since *P. lividus* TR61622_c8_g1_i2 transcript is not complete, it is possible that this protein is also targeted to the secretory pathway. It is predicted to be unstable (instability index), but thermo-stable over a wide temperature range (high aliphatic index) (Table S11). It presents a bias towards five amino acids: leucine (Leu), valine (Val), serine (Ser), glycine (Gly) and glutamine (Glu) (Table S11). A negative GRAVY and a theoretical pI around 5 (Table S11) indicate that this protein is hydrophilic and is negatively-charged upon secretion into seawater (pH = 8). Similar results were obtained for Arub-13 (sea star orthologue) and *S. purpuratus* Alpha-2-macroglobulin-like protein (Table S11). Several O-glycosylation sites are predicted for *P. lividus* Alpha-macroglobulin-like protein (Table S5), but the existence of predicted N- glycosylation sites cannot be excluded since the 5′ end of the gene is missing in the transcriptome. In fact, our pulldown results show that *P. lividus* Alpha-2-macroglobulin is most likely poorly glycosylated but possesses both O- (GalNAc residues) and N- (chitobiose) bound glycans. 

*P. lividus* Myeloperoxidase is an 829 amino-acid long protein, with a signal peptide followed by a peroxidase domain. Similar to *S. purpuratus* Myeloperoxidase (NCBI XP_787204.3) (Figure S5), both sequences have a bias towards leucine (Leu) and arginine (Arg). Predicted as unstable, not thermo-stable and hydrophilic, these proteins only differ in the theoretical pI. *P. lividus* myeloperoxidase seems to be negatively charged upon secretion (pI ~ 5). It is predicted to be poorly glycosylated, associated with N- and O- glycans. 

*P. lividus* Uncharacterized protein is a small protein, sharing 38.31% similarity with *S. purpuratus* Uncharacterized protein LOC115927989 (XP_030850287.1) (Figure S6). Both proteins have a predicted signal peptide, followed by a C-type lectin (CTL) domain, a carbohydrate-recognition domain. Mainly composed of leucine (Leu), serine (Ser), alanine (Ala) and valine (Val), both proteins have a predicted theoretical pI higher than eight, indicating that they are positively-charged upon secretion into seawater. *P. lividus* Uncharacterized protein was predicted as unstable, contrary to *S. purpuratus* Uncharacterized protein. Associated with N- and O-glycans (present in the four lectin pulldowns), and with no glycosylation prediction, our results can be explained by the threshold used during in silico analysis. Both TR46467_c1_g1_i1_6 and XP_030850287.1 contain a high cysteine content, having 14 (4.3%) and 10 cysteines (5%), respectively. Of these, four cysteine residues are highly conserved in the C-type lectin (CTL) domain, being involved in two disulfide bonds (Table 2). 

#### Nectin Variants

While protein variants have been extensively reported in marine adhesion, little information about their functional and molecular characteristics is available. Therefore, a closer analysis of Nectin sequences was performed, offering some insights into the molecular mechanisms regulating adhesion-related variants. 

All variants presented a high sequence similarity, with several repeat regions (correspondent to the six conserved tandemly repeated discoidin domains -FA58C) (Figure S7). These repeated domains were similar in each and between variants (47 to 55% identity). No inverted repeats were detected, but diagonal line discontinuities (Figure S7; see dotplot in which local regions of similarity correspond to diagonals) revealed small changes which led to the insertion/deletion of a few amino acids. Sequence alignment confirmed these results, showing eight amino acid substitutions between variants 2 and 3, and 13 substitutions between the new variant 3 and variant 1 (Figure S8). Eighteen substitutions were detected between all sequences, sixteen of them within the domains, seven of which were confirmed by MS/MS (present in the sequenced peptides). 

**Figure 6:**
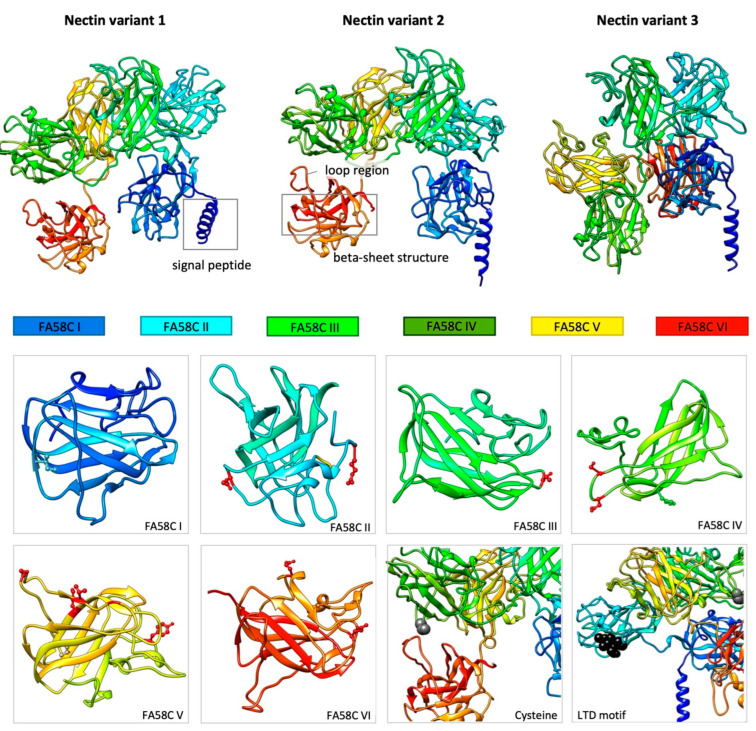
Comparative structural analysis of *Paracentrotus lividus* Nectin variants. Structural prediction of Nectin tertiary structure (first line) using Alphafold, with a close up on each of the six discoidin like domains (second and third line). Secondary structures are represented as arrows (beta-sheets), spirals (alpha-helix) and disorganized regions (random coils). Non-conservative amino acid substitutions are represented in red, while conservative substitutions maintain the color of the domain in which they occur. The cysteine residue responsible for Nectin homodimerization is indicated as a grey sphere and the LTD motif is identified as black spheres.

Protein tertiary structure is determined by the intramolecular noncovalent interactions established, depending on the chemical properties of the amino acid side chains. Due to its impact on protein function and activity, we detailed the physicochemical properties of the mutated amino acids and performed a 3D-structure prediction to understand the potential impact on protein structure (Figure 6). 

To date, only one homology-based fold structural prediction was performed [30] reporting a (1) single domain with a jelly-roll β-sheet of five and three strands packed against each other and (2) adjacent loops at the bottom of the β-barrel. We updated this prediction by using Alphafold, a recent IA system that considers local environments created by the amino acid scaffold, achieving high structure accuracy even for sequences with few homologous sequences [33,34]. To the best of our knowledge, this is also the first comparative structural analysis performed on *P. lividus* Nectin variants. 

This analysis showed a similar jelly-roll β-sheet structural arrangement in all FA58C domains (Figure 6). Due to an amino acid preference, these β-sheet-rich structures observed in all variants are a common feature of discoidin domains [35,36]. We also identified loop regions at the bottom of these structures in each domain, which were proposed as membrane contact points in the human coagulation factor V C2 domain [30]. Nectin variants 1 and 2 presented an irregular C-like tertiary structure. At this scale, the only visible alteration between these proteins was in FA58C II, which is (contrarily to variant 1) buried in the interior of variant 2. Variant 3 showed a clear conformational change with a spatial redistribution of the protein domains. With similar domains, molecular weights, physicochemical properties, and amino acid percentages (Table S11), we narrowed the analysis to amino acid substitutions (Table S12). Its side chain, depending on their physicochemical properties, can strongly impact protein structure and activity. 

Only 6 of the 18 observed substitutions were conservative, maintaining their physicochemical properties (Figure 6, Table S12). The internalization of FA58C II in variant 2 is consistent with the substitution of a polar amino acid with an uncharged side chain (asparagine) in variant 1 and 3, by a polar amino acid with a negatively charged side chain (aspartic acid). The substitution of polar-uncharged side chain with positively-charged side chain in both FA58C V and VI impacted their spatial organization, supporting this hypothesis [37]. FA58C IV has amino acid substitutions in the β-sheets involving threonine and serine modifications, possibly being implicated in post-translational modifications. Only glycine/serine substitution occurred more than once (FA58C IV and V), with glycine forming a highly hydrophobic core and serine being hydrophilic. Specific amino acid residues and their position in the polypeptide chain are the determining factors for which portions of the protein fold closely together and form its three-dimensional conformation. There is considerable evidence that hydrophobic interactions must play a major role in protein folding, where proteins with hydrophobic cores imply nonpolar amino acids to be sequestered from water [38]. This substitution bias, possibly explained by a single point mutation in serine first codon position (in one of the two disjoint sets codifying for serine) [39], does not seem responsible for variant 3 conformational change. In those positions, variant 2 presents the same amino acid and has a different structural arrangement.

A low cysteine content (1.1%) was observed in all variants, with: no cysteines in FA58C V, one in FA58C II, two in FA58C I and III and three in FA58C IV and VI. Based on protein folding, FA58C I cys43-cys49, FA58C III cys363- cys369, FA58C IV cys523-cys529 and FA58C VI cys927-cys956 are the only cysteine residues close enough to form the predicted S-S (disulfide) bridges. These results are in agreement with the homology-based prediction performed by [30]. Being consistently in the outer surface in all variants (Figure 6), cys605 seems to be responsible for Nectin homodimerization. All proteins have a LDT (integrin binding) motif, in the second discoidin domain, at the protein surface. The presence of all these features seems to indicate a preserved function between variants.

Post-translational modifications (PTMs) are also relevant for a proper protein function. Considering O-glycans, all variants are predicted to be glycosylated (Figure S8, Table S5). Small differences were observed regarding the generated proteolysis maps (enzymatic hydrolysis of a protein peptide bond by a specific protease), revealing that its specific mutations can define new cleavage sites (Figure S9). However, this bioinformatic analysis only considers the protein’s primary structure, not indicating if those cleavage sites are exposed in the tertiary or quaternary structure.

## 3. Discussion

### 3.1. Temporary Adhesives Are a Multi Glycoproteins Mixture

#### 3.1.1. High Molecular Weight Glycoproteins Are Involved in Sea Urchin Adhesion

To date, all characterized aquatic adhesives are composed of multiprotein complexes [27]. They are known to include large cohesive proteins and smaller adhesive proteins, some of which are glycoproteins, as reported for sea urchin *P. lividus* [17], sea star *A. gibbosa* [39] and flatworm *M. lignano* [8,21] being compartmentalized in the secretory granules. In these three aquatic temporary-attaching organisms, lectin histochemistry showed that glycoproteins were segregated in the outer rim of the adhesive secretory granules [8,17,21,40]. 

The use of adhesive organs-specific databases (e.g., sea urchin tube feet transcriptome [19]) conjugated with lectin assays and mass spectrometry, gave new insights regarding the molecular components involved in sea urchin adhesion. In the present work, we demonstrate that, of the previously-identified protein adhesive/cohesive candidates [19], at least five are glycoproteins. GSL II pulldowns pinpointed several glycoproteins containing GlcNAc, with an approximate molecular weight of 75, 135 and >245 kDa. WGA detected glycoproteins with up to two units of chitobiose, ranging from 35 to >245 kDa. LEL detected glycoproteins with up to four units of chitobiose only above 245 kDa. These results agree with previous work showing that by increasing lectin specificity in pulldowns (binding from GlcNAc monomers to a specific number of chitobiose units), it decreases the number of detected protein bands, a result supported by previous immunohistochemistry assays [17] showing that LEL produces the most specific labeling within the disc-adhesive epidermis, targeting the secretory granules. SBA-pulldowns have confirmed that sea urchin adhesive discs contain glycoproteins with N-acetylgalatosamine residues. The present study revealed the presence of several high molecular weight glycoproteins containing GalNAc (>135 kDa) in the eluted fraction, unlike previous reports of only two glycoproteins at 72 and 135 kDa [17]. The recurrent labelling of these two glycoproteins (72 and 135 kDa) with multiple lectins, targeting mainly the cytoplasm and microvilosities of abundant cells in the adhesive epidermis, led to the hypothesis that these proteins might be components of support cells instead of secretory cells [17]. Still, an indirect involvement in adhesion cannot be discarded since the knock-down of a support-cell-specific protein in the flatworm *M. lignano* produced a non-adhesive phenotype without influencing the production or secretion of the adhesive proteins [4,8]. It should be stressed that, despite their specificities, all lectins pulled-down high molecular weight glycoproteins (>245 kDa), which is in accordance with the identification of large putative adhesive/cohesive proteins such as *P. lividus* tube foot alpha-tectorin. 

#### 3.1.2. Adhesive/Cohesive Glycoprotein Candidates

According to our results, five glycoproteins containing GlcNAc and GalNAc residues seem to be involved in sea urchin adhesion: Nectin, Alpha-tectorin like protein, Myeloperoxidase, Alpha-2-macroglobulin-like protein, and two Uncharacterized proteins. 

The present work identified a third of *P. lividus* Nectin, confirming the involvement of adhesive proteins variants in sea urchin adhesion [29]. Adhesive protein variants have been also reported in hydra [41], barnacles [42], and in freshwater [43] and marine [44,45,46,47] mussels. Based on our comparative structural analysis, single nucleotide substitutions (SNPs) between variants show (1) a predominance towards non-conservative modifications at the apex of the loops between domains (a probable membrane contact point), (2) that it impacts protein tertiary structure and probably function, and (3) changes hydration on the protein surface (polar amino acids with different side chain electrical charges in different variants), known to play an important role in protein solubilization. Given that these SNPs are non-conservative and are predicted to alter protein conformation, it seems improbable that the existence of multiple Nectin sequences derive from nucleotide substitutions during DNA replication, due to high gene expression as previously hypothesized [29]. RNA splicing also does not explain the observed protein variation, since it would imply a change in sequence portions. Therefore, the existence of Nectin variants and their specific differences could be explained by RNA editing as a possible strategy to improve the adhesive versatility by producing multiple forms of an adhesive protein that can be fine-tuned to interact with surfaces with diverse chemical properties or even with different proteins [43,48,49,50]. Recent histological and molecular data support this hypothesis, showing that cement protein composition and their chemical properties in different barnacle species, may vary according with the attachment substratum [42]. A previous study seems to indicate the opposite by demonstrating that mussel foot protein 3 (Mfps-3) variants are not expressed differently depending on the substratum [44]. However, only 3 of the 35 variants were known at the time, so further studies need to be performed. Nevertheless, our results provide further evidences that Nectin is a key protein for sea urchin adhesion: (1) multiple variants are produced probably to cope with substrate chemistry; (2) being highly over-expressed in the tube feet adhesive discs and the secreted adhesive footprint [17,18]; (3) its expression is regulated depending on hydrodynamics and flow velocities [29,51], (3) possessing chitobiose residues probably corresponding to the glycans shown to be present in the outer rim of adhesive granules [17], and (5) being conserved in several sea urchins species representative of three orders and six families (embryonic Nectin-like proteins) [32].

Alpha-tectorin-like proteins can be linked to adhesive footprint cohesion and fibrous meshwork formation (already described for sea urchins adhesive [52]), by promoting protein multimerization into fibers [53], given its high cysteine content (known to be involved in strong disulfide bonds) and repetitive EGF-like and vWF domains). Being heavily glycosylated and containing glycan-binding lectin domains, sugar–protein interactions can also be involved in their non-covalent cross-linking with other components of *P. lividus* adhesive and/or of the disc cuticle [20]. 

Peroxidases can also contribute to the high cohesive strength of sea urchin adhesive secretions, attributed to the presence of proteins with significant amounts of cysteines [6,16]. Previous reports of peroxidase-like enzymes being highly expressed in sea urchin tube foot discs [18], in sea star adhesives [3] and in cnidaria attachment basal area [41] support their involvement in protein crosslink [54,55]. 

The involvement of protease inhibitors (Alpha-macroglobulin-like proteins) in sea urchin adhesion is in accordance with the current accepted enzymatic model [4,19,22]. Being reported in other aquatic temporary attaching organisms, such as barnacle cyprids (SIPC), sea stars (Arub-13, Spf-9), limpets (P- vulgata-5) and ascidians larvae (H2Y2X2) [4,53,56,57,58], protease inhibitors would provide control of the proteolytic action of the de-adhesive secretion and/or inactivate microbial proteases to protect the secreted adhesive from microbial degradation. The best-studied Alpha-macroglobulin-like adhesive protein is the settlement-inducing protein complex (SIPC) secreted by barnacle cyprid larvae. Used as a conspecific biochemical cue to induce the gregarious settlement of cyprids, it has been suggested to have a dual role as an adhesive [56,59,60] and protective protein [61]. Therefore, Alpha-2-macroglobulin-like protein in *P. lividus* adhesion might serve as a control mechanism to ensure the integrity of the adhesive secretion due to its inhibitory domains and/or to potentially mediate non-covalent cross-linking within the adhesive. Nevertheless, it is currently unknown whether these enzymes are produced in the de-adhesive cells or if they contribute to the de-adhesion process.

A combination of multiple EGF-like domains, von Willebrand factor type D domains (vWF), galactose-binding lectin domains (SUEL_Lectin) and discoidin-like domains (FA58C) seems to be recurrent in temporary attaching organisms, being reported in *P. lividus* (Alpha-tectorin and Nectin), sea stars (Spf1, 3, 4a and 4b), flatworms (Mlig-ap1 and -2, Mile-ap1 and Mile-ap2a/b), limpets (P-vulgata_1, 2, 3 and 4) and cnidaria. 

By providing a fine characterization of adhesive candidates involved in temporary sea urchin adhesion, our results reinforce the relevance of these functional domains and corroborate the existence of recurrent key elements in temporary marine adhesion.

## 4. Materials and Methods

### 4.1. Sampling and Animal Maintenance

*Paracentrotus lividus* were collected during low tide in Ericeira, Portugal (38°58′46.0″ N 9°25′20.4″ W). After collection, the animals were kept in re-circulating aquariums at 16–17 °C and 33‰.

### 4.2. Tube Foot Collection

Sea urchins (n = 30) were placed upside down in glass containers filled with artificial seawater (ASW). Their tube feet (n ≥ 30 per sea urchin) were sectioned by the base of the stem close to the test. Tube feet were dissected under a microscope with a razor blade, separating the adhesive discs from the non-adhesive stems. Adhesive discs were collected over eight consecutive days, immediately stored at −20 °C, and pooled for protein extracts (see Section 4.3). Sea urchins recover subsequently, returning to their natural habitat after sample collection. 

### 4.3. Protein Extracts

A dRIPA buffer was added to dissected discs (150 mM CaCl, 1.0% (*v*/*v*) Triton X-100, 0.5% (*w*/*v*) sodium deoxycholate, 0.1% (*v*/*v*) SDS, 50 mM Tris; pH 8.0) in a 1:2 proportion. A protease inhibitor cocktail (1:10,000) was added to the buffer to minimize proteolytic degradation. Then, mechanical lysis was performed using steel beads (2 mm diameter, Retsch) in a ball mill (MM 400 Retsch, Haan, Germany) and set to 30 H for 10 min. The resulting homogenate was centrifuged at 25,000× *g* (Hermle Z 323 K), for 10 min at 4 °C. The supernatant fraction was collected for further use.

### 4.4. Protein Quantification

The total protein concentration in the samples was determined using the Bradford colorimetric microplate assay (Bio-Rad, Hercules, CA, USA) and absorbances were obtained at a wavelength of 595 nm using a spectrophotometer (Amersham Imager 680 RGB, Cytiva Europe GmbH, Breisgau, Germany).

### 4.5. Lectin Pulldown

From tube feet disc protein extracts, glycoproteins-enriched fractions were obtained through lectin pulldowns (based on the reversible and highly selective binding of lectins to mono- and oligosaccharides). Four lectin-bound agarose beads were used (Vector Laboratories): GSL II, WGA and LEL (detecting different GlcNAc arrangements); SBA (GalNAc). Lectin-bound resins were equilibrated with 1 mL of TBS-T, supplemented with ions (20 mM Tris, 150 mM NaCl, 0.05% (*v*/*v*) Tween-20, 1 mM CaCl_2_, 1 mM MnCl_2_ pH 7.6) by continuous inversion (using a tube rotator) 4 times for 10 min. To prevent agarose bead loss, resins were centrifuged between washes at 15,000× *g* (Hermle Z 323 K) for 4 min at RT. Supernatant fractions were discarded. Equilibrated resins and samples were mixed overnight at 4 °C by continuous inversion. The mixture was centrifuged at 15,000× *g* for 4 min at 4 °C. The supernatant fraction containing unbound proteins (UB) was recovered and preserved at −20 °C. Lectin-bound resins were then washed with 1 mL of TBS-T supplemented with ions (20 mM Tris, 150 mM NaCl, 0.05% (*v*/*v*) Tween-20, 1 mM CaCl_2_, and 1 mM MnCl_2_ pH 7.6) 4 times for 10 min by continuous inversion. Between washes, the mixture was centrifuged at 15,000× *g* for 4 min at 4 °C. Four supernatant fractions, corresponding to the four washing steps (W), were recovered and preserved at −20 °C for later analysis. Then, 600 μL of glycoprotein eluting solution (Vector laboratories) was added to the mixture and incubated for 60 min at RT, with continuous inversion. The solution was then centrifuged at 15,000× *g* for 4 min at 4 °C and the supernatant fraction, corresponding to the eluted proteins (E), was recovered and stored at −20 °C until later analysis. A second elution step was performed, as described above, and pooled with the first eluted proteins (E) fraction.

All the samples, except for non-eluted proteins (NE), were concentrated by precipitation (see Section 4.7). The non-eluted fraction (NE) was only obtained later because it required boiling of the agarose beads with SDS-PAGE sample buffer. Beads were preserved at −20 °C until this step. Result specificity was assessed by inhibited lectin-bound agarose bead assays. Prior to overnight incubation, equilibrated resins were inhibited with a double elution step (750 μL ×2 of eluting sugar solution) by inversion for 2 h at RT.

### 4.6. Enzyme-Linked Lectin Assay (ELLA)

Wells of a 96-well microplate (Brand) were coated with 1 μg of protein per well at 4 °C and blocked with 0.5% polyvinyl alcohol in PBS for 2 h at RT. Polyvinyl alcohol was used as a blocking solution, since it prevents nonspecific interactions with the plate surface and does not interfere with the ability of lectins to interact with immobilized glycoproteins [62]. Subsequently, the wells were washed three times for 5 min with Tris-buffered saline, pH 7.6, containing 0.05% Tween 20 (TBS-T). Wells were then incubated with the lectins (1 μg/mL) prepared in TBS-T- ions (supplemented with 1 mM CaCl_2_, 1 mM MnCl_2_,1 mM MgCl_2_, 1 mM ZnCl_2_) for 1 h at 37 °C. Then, wells were washed as described above and incubated with horseradish peroxidase-conjugated streptavidin (Vector Laboratories, Newark, CA, USA) diluted 1:40,000 in TBS-T for 1 h at 37 °C. Then, wells were TBS-T washed, followed by incubation with TMB Substrate Solution (1- Step Ultra TMB Elisa; Thermo Fisher Scientific, Waltham, MA, USA) for 5 min at RT. The reaction was stopped with 2 M sulfuric acid and the absorbance was measured at 450 nm in a spectrophotometer (Amersham Imager 680 RGB, Cytiva Europe GmbH, Breisgau, Germany). For each lectin, eight replicate absorbances were obtained using discs extracts. Control reactions were performed by skipping protein addition to get a blank and using lectins saturated with their inhibitory monosaccharide (GlcNAcβ(1,4)GlcNAc and GlcNAc, for GSL II,WGA and LEL; GalNAc and Gal for SBA) or complex sugar mixture (E-5100 for GSL, WGA and LEL; E-2100 for SBA) (see Table S1). The statistical significance of differences between conditions was determined by a two-sample *t* test. *p* < 0.001 was considered to indicate a statistically significant difference (Table S2). Normality was checked using Shapiro–Wilks, as well as homoscedasticity using the F test or Levene’s test.

### 4.7. Protein Precipitation

The precipitation solution (10% (*w*/*v*) TCA, 0.07% (*v*/*v*) β-mercaptoethanol in water) was added to unbound, washing, and eluted fractions recovered during glycoproteins pulldowns. Each supernatant fraction was mixed for 5 min in a vortex mixer. All samples were precipitated for 90 min at 4 °C. Samples were then centrifuged at 6160× *g* (Hermle Z 323 K) for 20 min at 4 °C. All supernatant fractions were discarded. Pellets were washed with 1 mL of cold washing solution (0.07% (*v*/*v*) β-mercaptoethanol in acetone). Each precipitated sample/washing solution was vortexed for 5 min to mix. After mixing, samples were centrifuged at 6160× g for 20 min at 4 °C. A second and third wash were performed as described above. Pellets were dried overnight at 4 °C to allow acetone evaporation.

### 4.8. SDS Polyacrylamide Gel Electrophoresis

After casting, gels were placed inside an electrode assembly tank and afterward inside a tank. Both chambers were filled with Running Buffer 1× (25 mM Tris, 192 mM glycine, 0.1% (*v*/*v*) SDS, pH 8.3). Prior to electrophoresis, previously precipitated samples and the remaining agarose beads, were mixed with 30 μL of sample buffer (62.5 mM Tris-HCl pH 6.8, 25% (*v*/*v*) glycerol, 2% (*v*/*v*) SDS, 5% (*v*/*v*) β-mercapthoetanol and 0.01% (*w*/*v*) bromophenol blue) and boiled for 5 min at 96 °C. Agarose beads required an extra 4 min centrifugation at 15,000× *g* to obtain the supernatant fraction (non-eluted fraction—NE). Each gel was loaded with 5 μL of molecular weight markers (NZYBlue Protein marker, NZYTech) and 30 μL of each pulldown fraction. The run was performed in a mini-PROTEAN Tetra System (Bio-Rad, Hercules, CA, USA) at 50 V. After glycoproteins separation by molecular weight, gels were transferred to a PVDF membrane to assess for the presence of glycoproteins by lectin-blotting.

### 4.9. Lectin-Blotting

Glycoproteins were transferred to a polyvinylidene fluoride (PVDF) membrane and then blocked with 10 mM Tris-buffered saline pH 8 containing 0.05% Tween-20 and 3% BSA (TBS-T-BSA) at 4 °C overnight with constant rotation. Membranes were incubated for 1 h and 30 min with 1 of the 4 biotinylated lectins diluted at a concentration of 1 μg/mL in TBS-T-BSA supplemented with 1 mM CaCl_2_, 1 mM MnCl_2_,1 mM MgCl_2_, 1 mM ZnCl_2_ (TBA-T-BSA ions), followed by 5 washes in TBS-T. The membrane was then incubated with horseradish peroxidase-conjugated streptavidin (Vector Laboratories) diluted 1:5000 in TBS-T-BSA for 1 h in the dark, with rotation. After 5 washes in TBS-T, glycoproteins were visualized using an ECL immunoblot detection system (Amersham Imager 680 RGB, Cytiva Europe GmbH, Breisgau, Germany).

### 4.10. Proteomics Analyses

Two eluted fractions for each tested lectin (8 samples in total) were analyzed by mass spectrometry, each sample was composed of proteins extracted from 3 or more sea urchins recently collected from the natural environment. Samples for proteome analysis were first bound to 10 μL of Strataclean resin (Agilent, Stockport, UK), vortexed for 1 min, centrifuged, and the supernatant fraction was discarded. The Strataclean resin was then washed 3 times with 40 μL 25 mM ammonium bicarbonate. The beads with bound protein were treated with 0.05% (*w*/*v*) RapiGestTM (Waters, Wilmslow, UK) for 10 min at 80 °C, followed by reduction with 4 mM dithiothreitol for 10 min at 60 °C, alkylation with 14 mM iodoacetamide for 30 min at RT, and finally, digested with 0.5 μg of trypsin in two steps (4 h at 37 °C, followed by overnight at 37 °C with agitation to keep beads suspended). Then, samples were acidified with 0.5% (*v*/*v*) trifluoroacetic acid for 45 min at 37 °C, centrifuged and 2.5 μL of clarified supernatant fraction was injected onto a reversed-phase nanoflow column, developed over a 50 min gradient. Eluted peptides were analyzed by tandem mass spectrometry on an Orbitrap Q Exactive HF (Thermo Fisher Scientific, Altringham, UK) mass spectrometer, using conditions previously published [63]. MS/MS data were searched for adhesive protein candidates against a database composed of the six open reading frames (ORFs) of *P. lividus* tube foot transcriptome [18] using Mascot (version 2.7.0, Matrix Science, London, UK). The peptide mass tolerance was set to 10 ppm, and fragment mass tolerance was set to 0.01 Da. Carbamidomethyl cysteine was set as a fixed modification, and oxidized methionine as variable modification. The false discovery rate (FDRs) was set to 1%. Protein hits based on one unique peptide were excluded from subsequent analysis. Of these, only hits with more than one unique peptide were analyzed [64,65].

### 4.11. In Silico Analysis

Sequence similarity was analyzed with Basic Local Alignment Search Tool (BLAST) [66] from (NCBI) and (UniProt). Similar sequences were aligned using Cobalt Alignment Tool from NCBI. NCBI Conserved Domain Databases [67] and InterPro [68] were used to domain prediction. The presence of a signal peptide, a feature of secreted proteins, was predicted using SignalP 5.0 [69]. The amino acid composition, total number of charged residues, instability index, aliphatic index and hydrophobicity (GRAVY index) were predicted with ProtParam tool from Expasy [70]. The theoretical isoelectric point and molecular weight of each protein were predicted using Compute pI/MW tool from Expasy. Putative N- and O-glycosylation sites were predicted using NetNGlyc 1.0 and NetOGlyc 4.0 [71] considering a threshold of >0.5). Protein three-dimensional structure was performed using Alphafold [33]. Structural alignment, structure edition and protein visualization were performed with UCSF Chimera [72]. Dot matrix plots were performed with EMBOSS Dotmacher (considering the default settings) [73]. 

## 5. Conclusions

To date, all characterized aquatic adhesives are composed of multiprotein complexes [27]. They are known to include large cohesive proteins and smaller adhesive proteins, some of which are glycoproteins that segregate in the outer rim of the adhesive secretory granules [8,17]. This work demonstrates that sea urchin temporary adhesion is no exception. We report the presence of five adhesive protein candidates conjugated with N-Acetylglucosamine and N-Acetylgalactosamine residues: four high molecular weight proteins—Nectin, Alpha-2-macroglobulin-like protein 1, Myeloperoxidase and Alpha-tectorin like protein and one low molecular weight Uncharacterized protein. The identification and characterization of a third Nectin variant suggest a relevant role for protein variants in sea urchin adhesion. By providing a deeper characterization of sea urchin adhesive/cohesive glycoproteins, this work further unravels the molecular composition of its adhesive, crucial to the biomimetic design process.

## Figures and Tables

**Table 1:** Candidate adhesive/cohesive proteins list derived from MS/MS and BLAST analysis. Abbreviations: COV: sequence coverage (%; values obtained in the two replicates); MW: molecular weight (kDa); pI: isoelectric point.

Transcript	Pjeta et al. 2020 (BLAST)	2022 (BLAST)	GSL II	WGA	LEL	SBA	Unique Peptides	MW	pI
			COV	COV	COV	COV			
TR60905_c1_g1_i1_5	Nectin variant 2 precursor [*Paracentrotus lividus*]	Nectin 2 *[Paracentrotus lividus*]	39 | 35	41 | 34	37 | 31	39 | 35	40	108.24	5.98
TR63383_c2_g1_i1_5	PREDICTED: alpha-tectorin [*Strongylocentrotus purpuratus*]	Uncharacterized protein LOC100892803 [*Strongylocentrotus purpuratus*]	9 | 6	10 | 0.5	8 | 4	15 | 12	21	199.71	4.81
TR46688_c0_g1_i1_6	PREDICTED: uncharacterized protein LOC100892803 [*Strongylocentrotus purpuratus*]		12 | 10	7 | 5	11 | 11	13 | 5	9	74.09	5.43
TR57217_c2_g1_i1_5	PREDICTED: myeloperoxidase *[Strongylocentrotus purpuratus*]	Myeloperoxidase [*Strongylocentrotus purpuratus*]	5 | 5	11 | 13	3 | 4	8 | 8	9	94.02	4.94
TR61622_c8_g1_i2_4	PREDICTED: α-2-macroglobulin-like protein 1 isoform X1 [*Strongylocentrotus purpuratus*]	α-2-macroglobulin-like protein [*Strongylocentrotus purpuratus*]	10 | 10	11| 10	11 | 10	14 | 11	29	157.63	5.15
TR46467_c1_g1_i2_6	N/A	Uncharacterized protein LOC115927989 [*Strongylocentrotus purpuratus*]	25 | x	25 | x	14 | 25	19 | 27	8	20.81	8.86

**Table 2:** Glycosylated protein candidates proposed as relevant for *Paracentrotus lividus* adhesion. Abbreviations: A2M, alpha macroglobulin domain; C8, cysteine-rich domain; CTL, C-type lectin domain; EGF, EGF-like calcium-binding domain; FA58C, discoidin domains; N: N-glycosylation; O: O-glycosylation; SUEL lectin, galactose-binding lectin domains; TIL, trypsin inhibitor-like; and vWD, von Willebrand domain.

Transcript	Sequence ID	Sequence Completeness	Start/Stop Codon	Signal Peptide	Cysteine Content	Predicted Glycosylation	Conserved Domains	Homologous Proteins of Interest
TR60905_c1_g1_i1_5	Nectin variant 3	full length	yes/yes	yes	1.1%	O,N	FA58C	Nectin
TR63383_c2_g1_i1_5	α-tectorin like	5’ missing	no/yes	no	5.1%		SUEL-Lectin, EGF, vWD, C8, TIL	---
TR46688_c0_g1_i1_6		5’ & 3’ missing	no/no	no	7.1%			α-tectorin, Spf1
TR57217_c2_g1_i1_5	myeloperoxidase	full length	yes/yes	yes	3.3%		Peroxidase	Myeloperoxidase
TR61622_c8_g1_i2_4	α-2-macroglobulin-like	5’ missing	no/yes	no	2.1%		A2M_N_2, A2M, A2M_2, A2M-recp	α-2-macroglobulin-like protein 1
TR46467_c1_g1_i2_6	uncharacterized protein	full length	yes/yes	yes	4.3%	O	CTL	---

## Data Availability

The sequences were deposited at GenBank with the following accession numbers: Nectin (OP810558); α-2-macroglobulin like protein (OP810559); Myeloperoxidase (OP810560); Uncharacterized Protein (OP810561); α-tectorin like protein (OP810562 and OP810563).

## Supplementary Materials

The following supporting information can be downloaded at: https://www.mdpi.com/article/10.3390/md21030145/s1, Table S1: Enzyme-linked lectin assay absorbance values; Table S2: Enzyme-linked lectin assay statistical analysis; Table S3: Identification of lectin- pulldown proteins; Table S4: Candidate adhesive proteins proposed by [19] Table S5: Adhesive candidate proteins glycosylation; Table S6: Nectin variant 3 unique peptides; Table S7: Alpha-macroglobulin unique peptides; Table S8: Alpha-tectorin like unique peptides; Table S9: Myeloperoxidase unique peptides; Table S10: Uncharacterized protein unique peptides; Table S11: Molecular features of adhesive and/or cohesive glycoproteins identified in *Paracentrotus lividus* tube feet; Table S12: Amino-acid substitutions between *Paracentrotus lividus* Nectin variants; Figure S1: Proteomic workflow of the present study; Figure S2: Global protein composition by functional groups of *Paracentrotus lividus* disc proteins pulldown with lectin-bounded agarose beads; Figure S3: Alpha-2-macroglobulin like sequence alignment; Figure S4: Alpha-tectorin like sequence alignment; Figure S5: Myeloperoxidase sequence alignment; Figure S6: Uncharacterized protein sequence alignment; Figure S7: Comparison of Nectin variants; Figure S8: Nectin sequence alignment; Figure S9: Proteolysis map of Nectin variants.
