# Supplementary material for: Glycoproteins Involved in Sea Urchin Temporary Adhesion
Source: Mar Drugs. 2023 Feb 24;21(3):145. doi: 10.3390/md21030145 (PMC10057474; doi:10.3390/md21030145)
Supplement: Supplementary file 1 [file marinedrugs-21-00145-s001.zip › Supplementary figures.pdf]

marine drugs

Supplementary Material

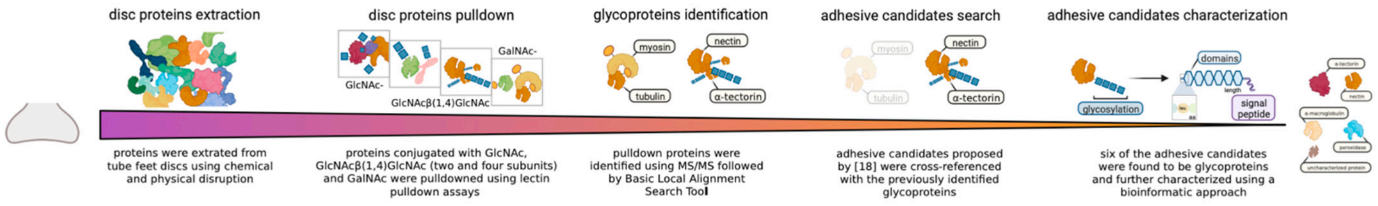

Figure S1. Proteomic workflow of the present study. Schematic representation of the study workflow. Sample collection days: 8 (N=3 sea urchins); tandem MS and pull-downs (N=2).

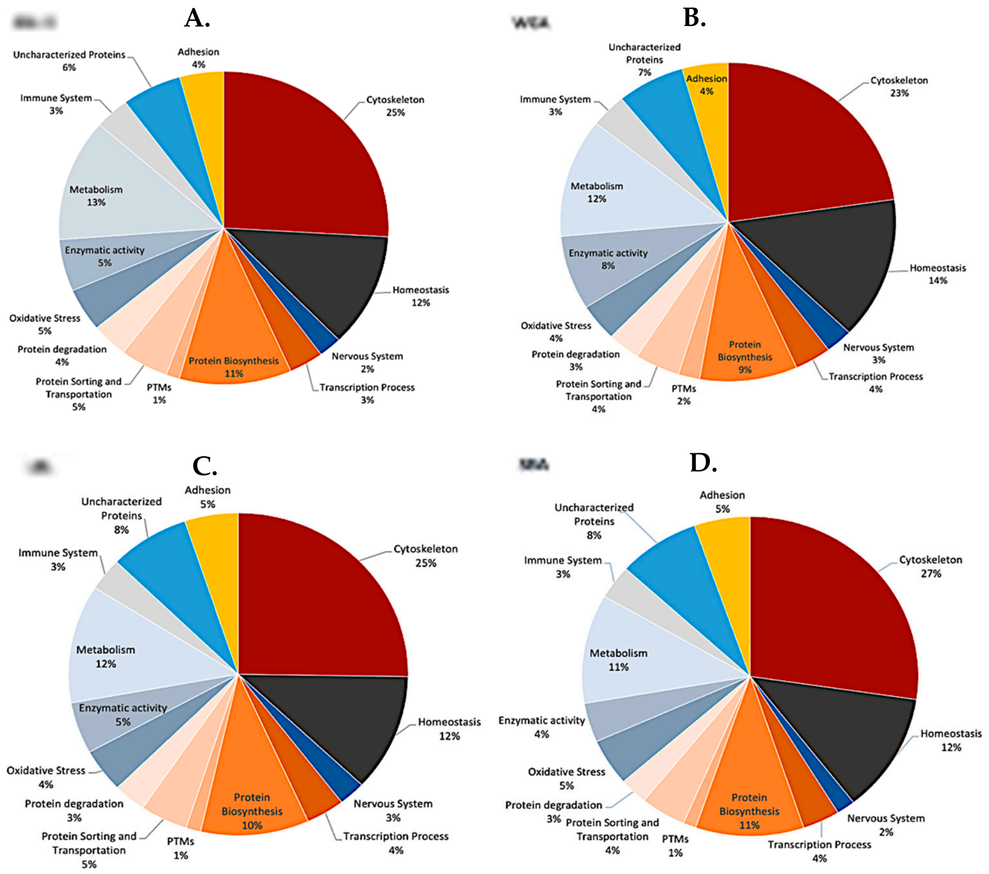

Figure S2. Global protein composition by functional groups of *Paracentrotus lividus* disc proteins pulldown with lectin-bounded agarose beads. GSL II was used to detect the presence of N-acetylglucosamine; WGA and LEL to detect chitobiosis and SBA to detect N-acetylgalactosamine conjugated with proteins (N=2). Abbreviations: GSL II, *Griffonia simplicifolia* lectin II; LEL, *Lycopersicon esculentum* lectin;

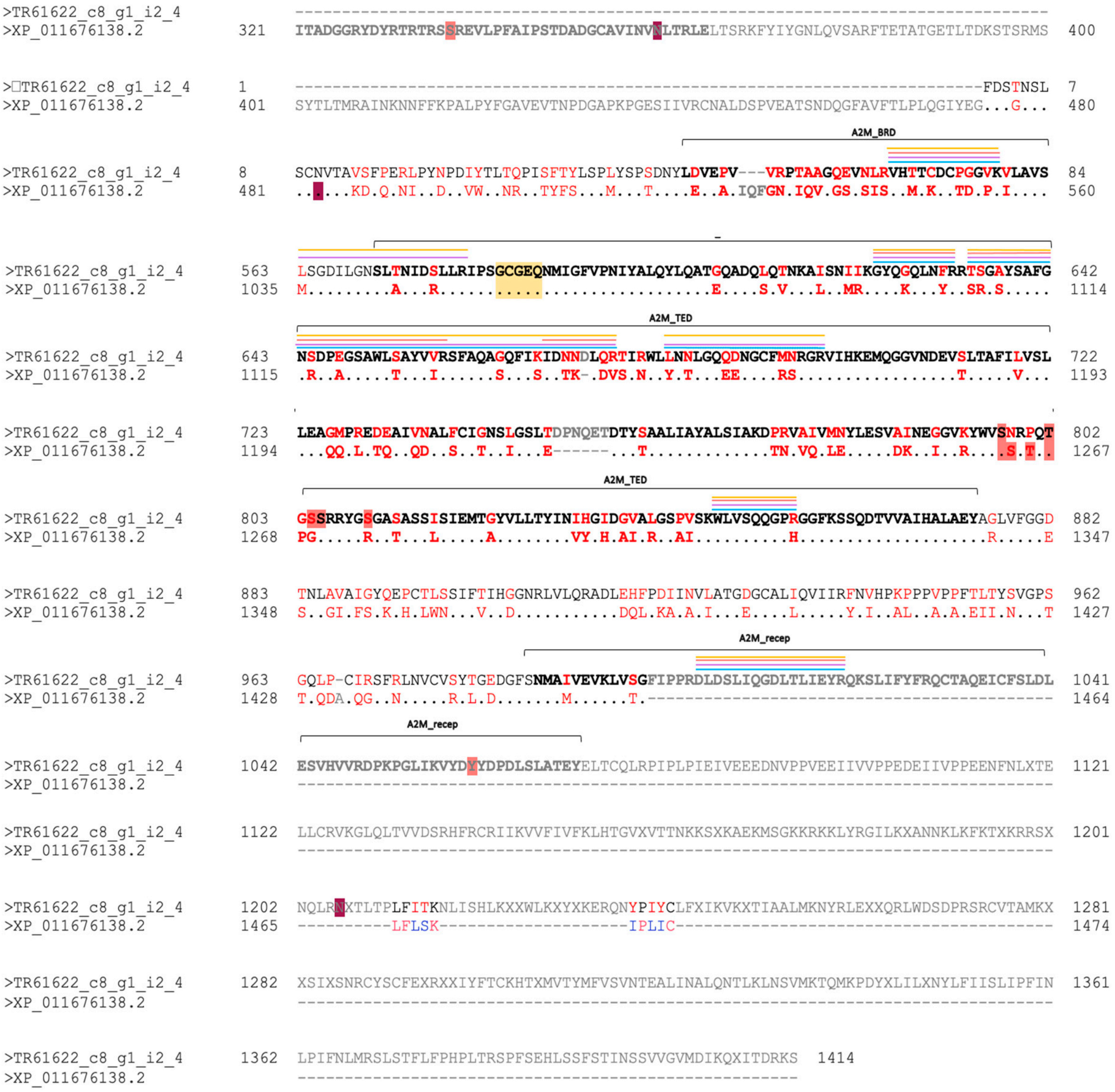

Figure S3. Alpha-2-macroglobulin like sequence alignment. Sequence alignment of *Paracentrotus lividus*alpha-2-macroglobulin and sea urchin *Strongylocentrotus purpuratus* alpha-2-macroglobulin homologous protein. Between sequences, black dots represent equal amino acids, red letters different amino acids in the same position and dash missing amino acids in the aligned sequence. Identified peptides by MS/MS (dashed over the sequences) validated N- (in purple) and O--glycosylation sites. Peptides were found in several lectin-pulldowns specifically GSL II (blue dash), WGA (pink), LEL (purple) and SBA (orange).

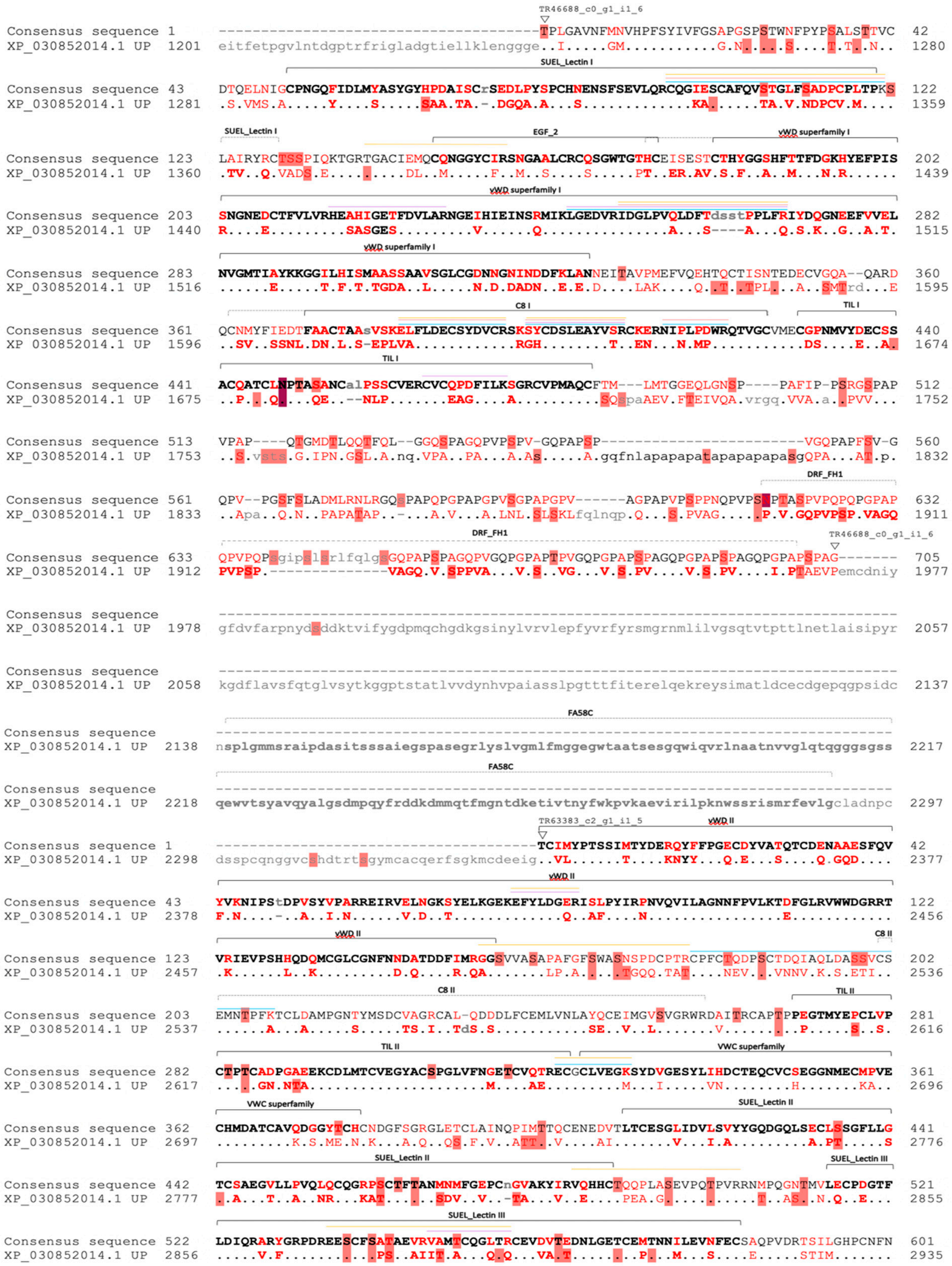
TR46688_c0_g1_i1_6
Consensus sequence
XP_030852014.1 UP
SUEL_Lectin I
EGF_2
vWD superfamily I
C8 I
TIL I
DRF_FH1
FA58C
TR63383_c2_g1_i1_5
vWD II
C8 II
TIL II
VWC superfamily
SUEL_Lectin II
SUEL_Lectin III

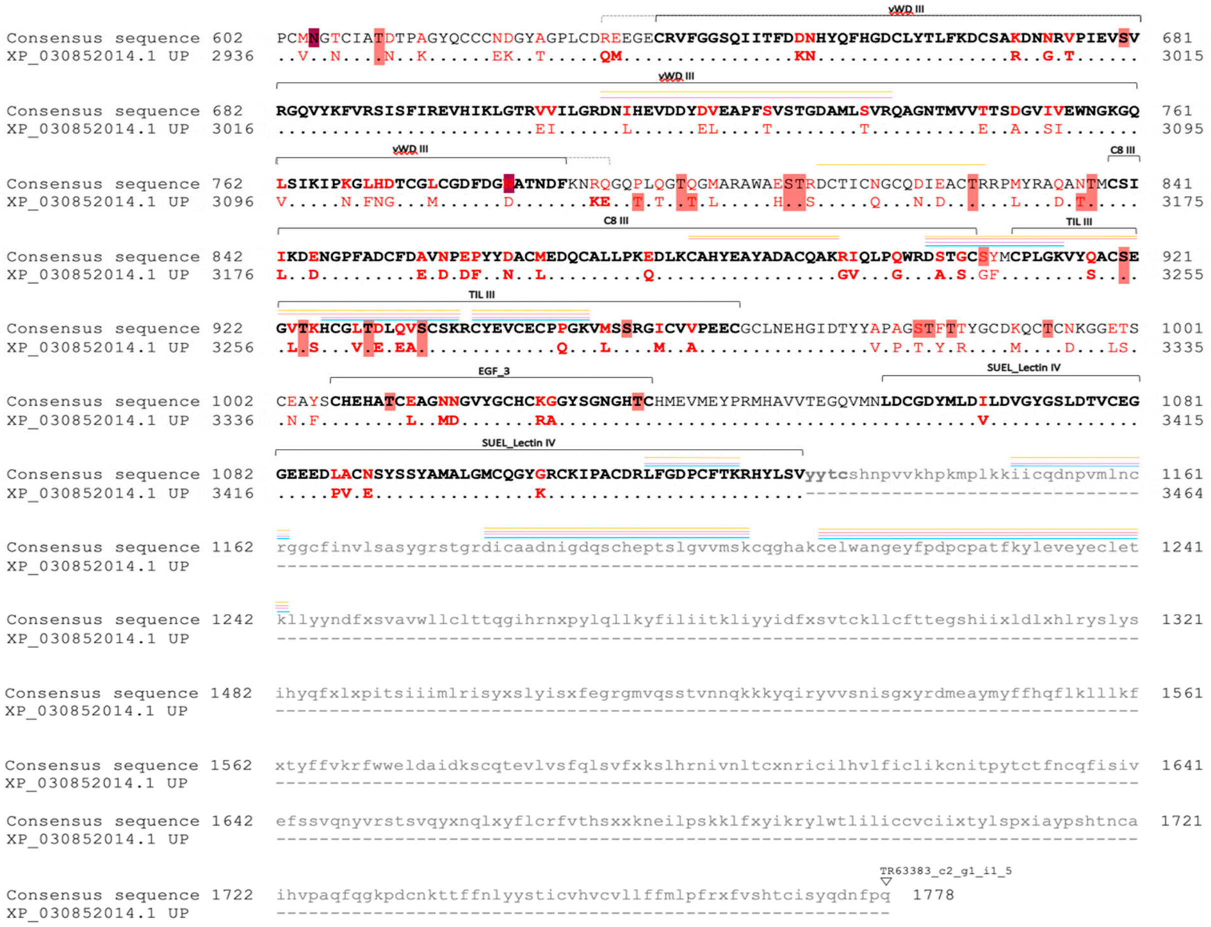

Figure S4. Alpha-tectorin like sequence alignment. Sequence alignment of *Paracentrotus lividus* alpha-tectorin and sea urchin *Strongylocentrotus purpuratus* uncharacterized protein LOC100892803 homologous protein. Between sequences, black dots represent equal amino acids, red letters different amino acids in the same position and dash missing amino acids in the aligned sequence. Identified peptides by MS/MS (dashed over the sequences) validated N- (in purple) and O--glycosylation sites. Peptides were found in several lectin-pulldowns specifically GSL II (blue dash), WGA (pink), LEL (purple) and SBA (orange).

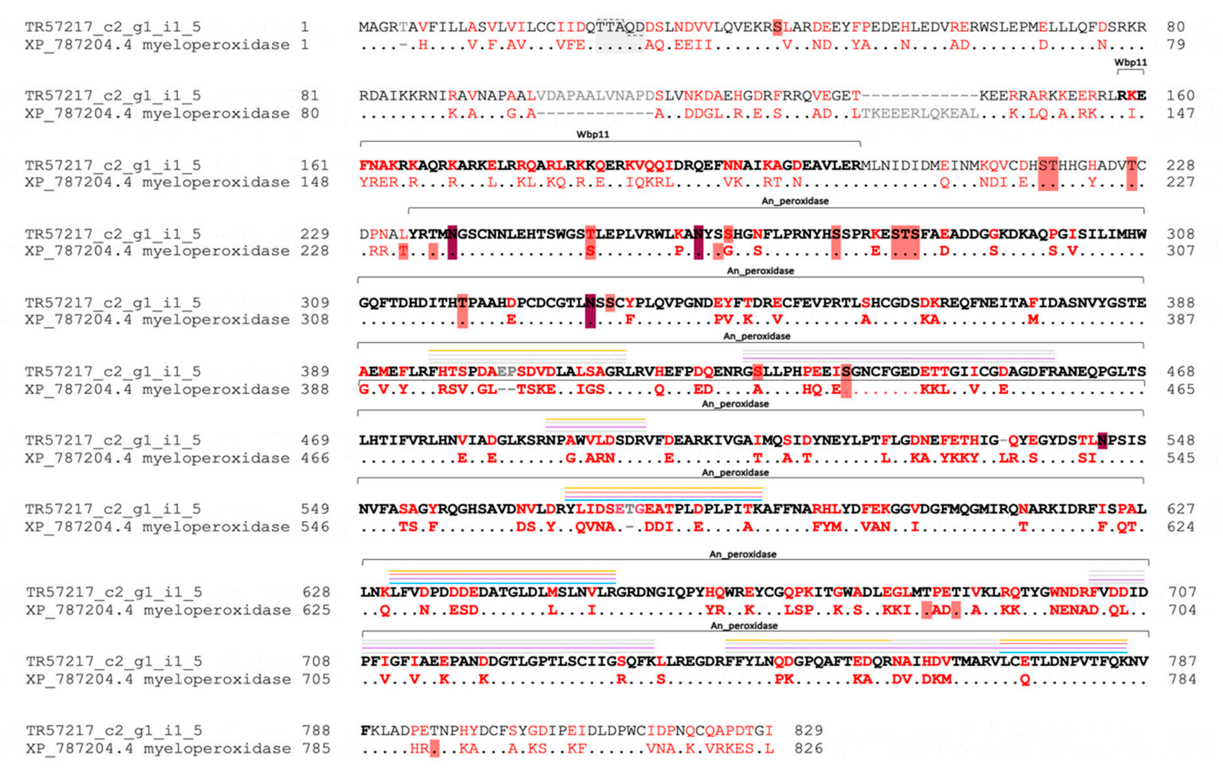

Figure S5. Myeloperoxidase sequence alignment. Sequence alignment of *Paracentrotus lividus* and sea urchin *Strongylocentrotus purpuratus* myeloperoxidase homologous proteins. Between sequences, black dots represent equal amino acids, red letters different amino acids in the same position and dash missing amino acids in the aligned sequence. Identified peptides by MS/MS (dashed over the sequences) validated N- (in purple) and O--glycosylation sites. Peptides were found in several lectin-pulldowns specifically GSL II (blue dash), WGA (pink), LEL (purple) and SBA (orange).

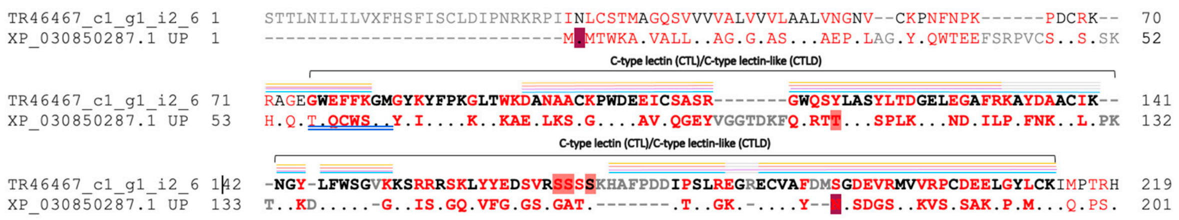

Figure S6. Uncharacterized protein sequence alignment. Sequence alignment of *Paracentrotus lividus* and sea urchin *Strongylocentrotus purpuratus* uncharacterized proteins homologous proteins. Between sequences, black dots represent equal amino acids, red letters different amino acids in the same position and dash missing amino acids in the aligned sequence. Identified peptides by MS/MS (dashed over the sequences) validated N- (in purple) and O--glycosylation sites. Peptides were found in several lectin-pulldowns specifically GSL II (blue dash), WGA (pink), LEL (purple) and SBA (orange).

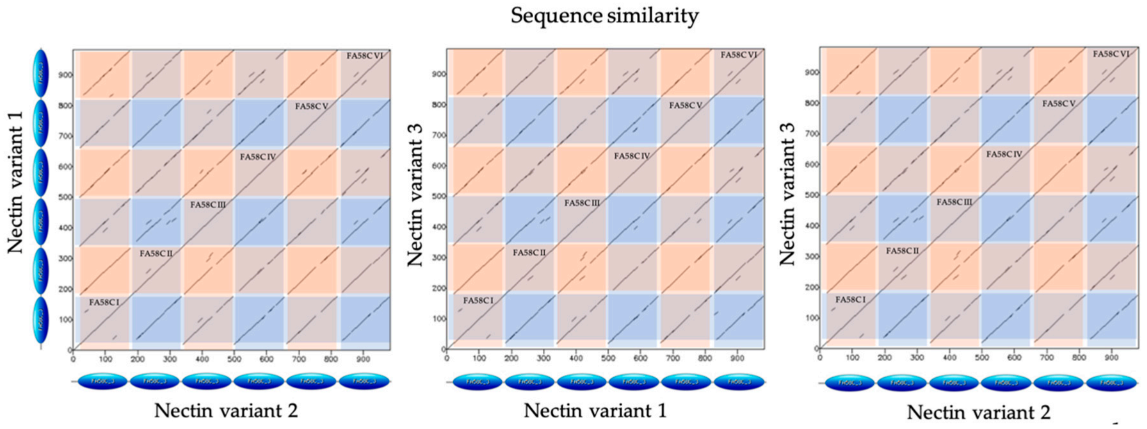

Figure S7. Comparison of Nectin variants. Graphical representation of the similarity regions between two protein sequences using EMBOSS Dotmatcher. Local regions of similarity correspond to diagonals in the dotplot. Structural variations are represented as gaps on the diagonal lines (deletion and insertions) or duplication of a sequence segment towards one of the axis (duplication).

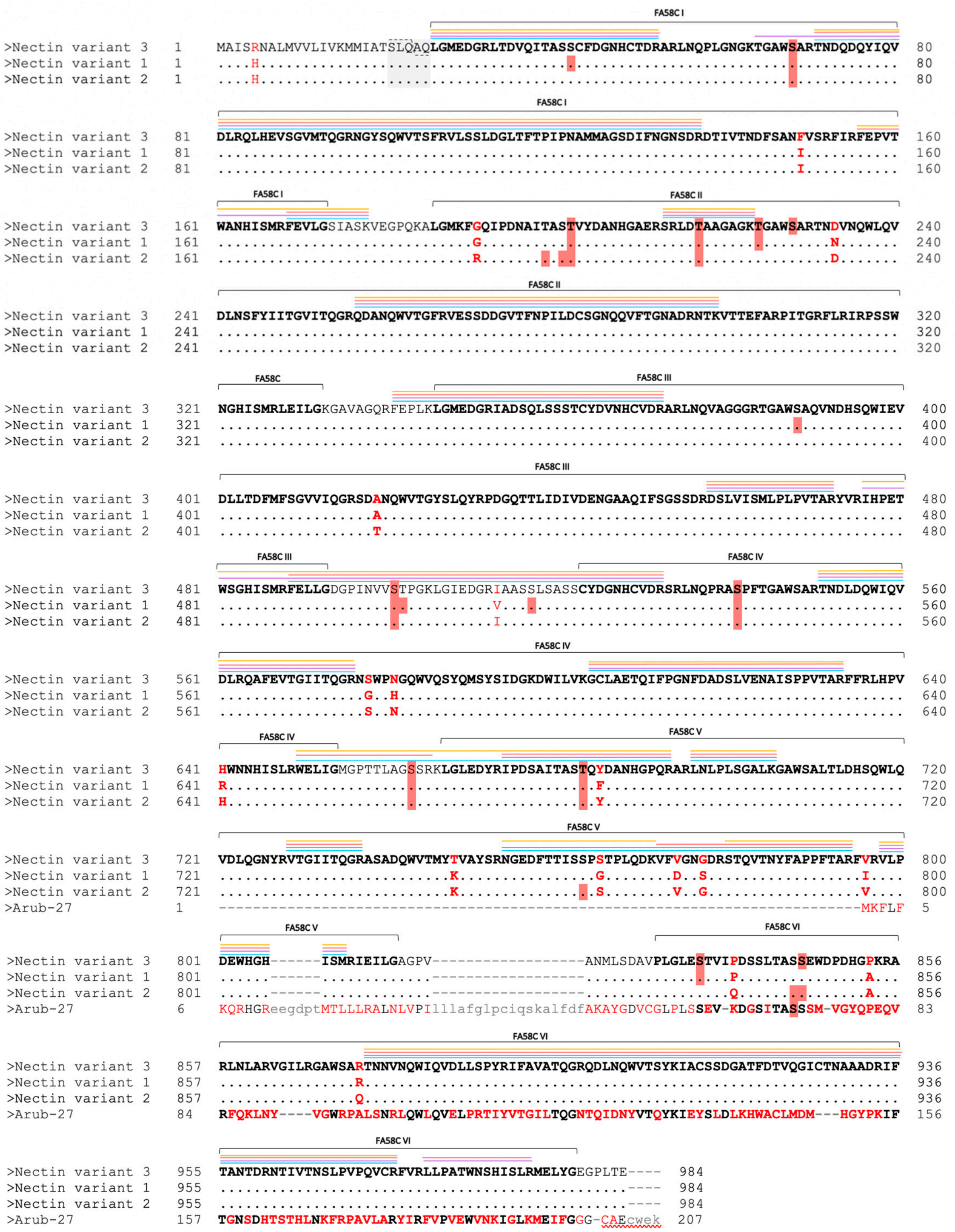

Figure S8. **Nectin sequence alignment.** Sequence alignment of *Paracentrotus lividus* Nectin variants and sea star *Asteria rubens* Arub-27. Between sequences, black dots represent equal amino acids, red letters different amino acids in the same position and dash missing amino acids in the aligned sequence. Identified peptides by MS/MS (dashed over the sequences) validated N- (in purple) and O--glycosylation sites. Peptides were found in several lectin-pulldowns specifically GSL II (blue dash), WGA (pink), LEL (purple) and SBA (orange).

Nectin variant 1 proteolysis map

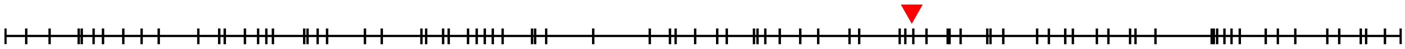

Nectin variant 2 proteolysis map

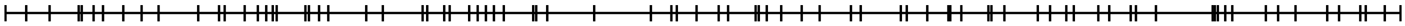

Nectin variant 3 proteolysis map

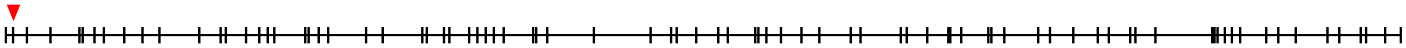

Figure S9. Proteolysis map of Nectin variants. Sites predicted to be cleaved by trypsin were performed with proteolysis mapping tool. Cleavage sites are identified by black lines, while differences between sequences are enphasize as a red arrow.
